# Salivary RANKL and OPG gene expression quantification during intermaxillary elastic traction in orthodontic patients^☆^

**DOI:** 10.1016/j.mex.2026.103994

**Published:** 2026-06-06

**Authors:** Danusha Siva Dharma, Siti Hajjar Nasir, Muhamad Ashraf Rostam, Kumeran Mohan, Noraini Abu Bakar

**Affiliations:** a Department of Orthodontics, Kulliyyah of Dentistry, International Islamic University Malaysia, Kuantan, Pahang, Malaysia; b Department of Biomedical Science, Kulliyyah of Allied Health Science, International Islamic University Malaysia, Kuantan, Pahang, Malaysia

**Keywords:** Orthodontic tooth movement, OTM biomarkers, RT-qPCR, Orthodontic elastic, Clinical orthodontics

## Abstract

This protocol describes a non-invasive workflow for quantifying salivary RANKL and OPG gene expression in orthodontic patients undergoing intermaxillary elastic traction. Unstimulated whole saliva was collected by passive drooling at three predefined time points: baseline before elastic initiation (T0), 24 h (T1), and 7 days (T2), from 30 female orthodontic patients allocated to Class I fixed appliance-only, Class II elastics, and Class III elastics treatment groups. Salivary pellets obtained by sequential centrifugation and physiological saline washing were subjected to column-based total RNA extraction, spectrophotometric quality control, and first-strand cDNA synthesis. Relative gene expression of RANKL and OPG was quantified by RT-qPCR using β-actin as the internal reference gene. Fold-change expression was calculated relative to individual patient baseline values. Group and temporal comparisons were performed using linear mixed models with Bonferroni-corrected pairwise contrasts. This protocol provides sufficient procedural detail for direct replication in prospective cohort investigations of mechanically induced molecular events in orthodontic treatment.

• A complete clinical workflow using non-invasive saliva collection for column-based RNA extraction and RT-qPCR amplification following MIQE guidelines.
• Optimised pre-analytical and analytical steps.
• A prospective three-group design enables isolation of vector-specific gene expression changes during fixed appliance mechanics.

## Graphical abstract

**Figure:**
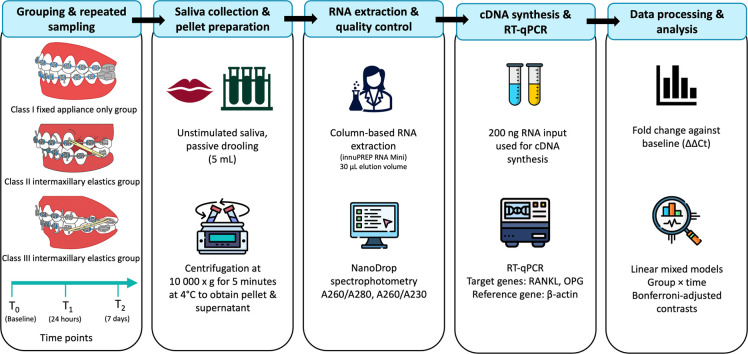

## Specifications table

**Table:** 

**Subject area**	Medicine and Dentistry
**More specific subject area**	Orthodontics; Molecular Biology
**Name of your protocol**	Salivary RANKL and OPG gene expression quantification protocol using RT-qPCR during orthodontic intermaxillary elastic traction
**Reagents/tools**	innuPREP RNA Mini Kit 2.0 (IST Innuscreen GmbH, Germany); SensiFAST™ cDNA Synthesis Kit BIO-65,054 (Meridian Bioscience, USA); SensiFAST™ SYBR® No-ROX Kit BIO-98,005 (Meridian Bioscience, USA); NanoDrop™ 1000 spectrophotometer (Thermo Fisher Scientific, USA); CFX96 Touch Real-Time PCR Detection System (Bio-Rad, USA); CFX Maestro Software v5.3 (Bio-Rad, USA); Hettich Mikro 220R refrigerated microcentrifuge (Andreas Hettich GmbH, Germany); IBM SPSS Statistics v25.0; GraphPad Prism v10.0.0.
**Experimental design**	Prospective cohort study; three malocclusion groups (Class I, II, III; n = 10/group); 30 female participants aged < 40 years; three saliva collection time points (T0 baseline, T1 24 h, T2 7 days); RT-qPCR for RANKL and OPG; ΔΔCt method with β-actin normalisation; linear mixed model statistical analysis
**Trial registration**	Nil
**Ethics**	Ethical approval obtained from the IIUM Research Ethics Committee (IREC) (IREC 2024–032) prior to the commencement of patient recruitment and data collection. The study was conducted in full accordance with the ethical principles outlined in the Declaration of Helsinki for research involving human patients. Written informed consent was obtained from all patients prior to enrolment. For patients below 18 years of age, written informed consent was obtained from a parent or legal guardian in addition to the participant’s consent. Confidentiality of the patient’s information was strictly maintained throughout the study. All patients’ data and biological samples were coded using unique identification numbers, and no personal identifiers were used during data analysis or reporting.
**Value of the Protocol**	• Provides a complete, end-to-end clinical RT-qPCR protocol for salivary RANKL and OPG gene expression during orthodontic intermaxillary elastic traction, adaptable to any longitudinal orthodontic biomarker study design. • Addresses the critical pre-analytical challenge of salivary RNA extraction by incorporating a validated three-cycle pellet-wash protocol to remove mucin-derived PCR inhibitors, enhancing RNA purity and downstream amplification reliability. • Enables controlled isolation of elastic-type-specific molecular responses through a three-group prospective design incorporating a fixed appliance comparator, facilitating rigorous between-group biomarker comparisons in a clinical setting.

## Background

Orthodontic tooth movement (OTM) is driven by mechanically induced alveolar bone remodelling, a process fundamentally regulated by the receptor activator of nuclear factor-κB ligand (RANKL) and its decoy receptor, osteoprotegerin (OPG). RANKL promotes osteoclastogenesis and bone resorption, while OPG acts as its endogenous inhibitor; the RANKL/OPG ratio is widely regarded as a molecular index of the net resorptive or formative balance at the bone-ligament interface. Both mediators are detectable in whole unstimulated saliva, which offers a non-invasive, repeatable biological matrix well-suited to longitudinal clinical sampling during orthodontic treatment.

Intermaxillary elastic traction is among the most clinically prevalent adjuncts in fixed appliance orthodontic treatment, used routinely to correct sagittal discrepancies in Class II and Class III malocclusion. Despite widespread use, the molecular biological response to this specific force modality remains incompletely characterised. The existing biomarker literature is dominated by studies of forces from archwires [1] and brackets [2]; comparative data from intermaxillary elastics applied across two arches and two distinct force vectors are limited. This evidence gap constrains our ability to predict inter-individual variation in treatment response and to develop biologically informed benchmarks for monitoring elastic traction efficacy.

A critical methodological challenge in salivary RT-qPCR is the low yield and variable quality of RNA extracted from unstimulated whole saliva, which contains mucins, cellular debris, and microbial contaminants that co-purify with nucleic acids and inhibit downstream enzymatic reactions [3,4]. Reliable pre-analytical processing is therefore essential for reproducible gene expression data. Additionally, the use of validated, constitutively stable reference genes and adherence to Minimum Information for Publication of Quantitative Real-Time PCR Experiments (MIQE) guidelines are required to ensure analytical rigour and inter-study comparability.

The present protocol is distinguished from previous orthodontic biomarker studies by integrating an unstimulated whole-saliva RT-qPCR approach with a mechanics-specific three-group prospective design. Unlike most salivary RANKL and OPG studies, which have measured protein concentrations using ELISA or multiplex immunoassays [1,5], this protocol quantifies RANKL and OPG gene expression from salivary pellets using MIQE-aligned RT-qPCR procedures [6], addressing the recognised difficulty of detecting low-abundance salivary RANKL by conventional immunoassay methods [1]. It also focuses specifically on intermaxillary elastic traction, a clinically common but biomarker-understudied force system that differs from continuous fixed appliance mechanics because of its intermittent, patient-dependent force delivery and progressive force decay [7]. By incorporating Class I fixed appliance-only patients as a comparator alongside Class II and Class III elastic-traction groups, the protocol enables elastic-related transcriptional responses and opposing sagittal force vectors to be examined within a single methodological framework, which has not been reported in previous salivary RANKL/OPG protocols.

Beyond its methodological novelty, the protocol is beneficial for both patients and the orthodontic research community. For patients, unstimulated whole saliva collection offers a simple, non-invasive sampling method that avoids the discomfort and technical demands of site-specific GCF collection, making repeated monitoring more acceptable during longitudinal orthodontic treatment, particularly in younger patients. For researchers and clinicians, the protocol provides a standardised framework for generating comparable salivary molecular data across future studies, supporting more consistent evaluation of biological responses during OTM. With further validation in larger cohorts linked to clinical outcomes, such as tooth movement rate, treatment efficiency, and adverse effects, this approach may contribute to the development of non-invasive biomarker monitoring strategies for biologically informed orthodontic care.

The present protocol was developed and validated within a prospective cohort study at the Orthodontic Clinic, Kulliyyah of Dentistry, International Islamic University Malaysia (IIUM), involving female orthodontic patients allocated to Class I (fixed appliance comparator), Class II, and Class III malocclusion groups. Three biologically relevant time points: baseline (T_0_), 24 h (T_1_), and 7 days (T_2_) of elastic traction were selected to capture the initial mechanotransduction phase and the osteoclastogenic cascade phase of OTM. This protocol description provides all pre-analytical and analytical steps necessary for direct replication of this study design or adaptation to related orthodontic biomarker research.

## Description of protocol

### Study design and patient eligibility

A quantitative prospective cohort design was employed. Participants were allocated to three groups according to their malocclusion classification and planned orthodontic treatment:

• Class I malocclusion — fixed appliance only (non-extraction; no intermaxillary elastic traction; fixed appliance control group).
• Class II malocclusion — at least 2 maxillary premolar extractions and received Class II intermaxillary elastic traction (Intervention Group 1).
• Class III malocclusion — at least 2 mandibular premolar extractions and received Class III intermaxillary elastic traction (Intervention Group 2).

**Inclusion criteria:**

• Female patients aged < 40 years undergoing fixed appliance therapy (MBT prescription, 0.022 × 0.028-inch slot).
• Completed levelling and alignment phase with progression to 0.019 × 0.025-inch stainless steel working archwires in both arches.
• BMI within normal range (18.5–24.9 kg/m²).
• Malocclusion confirmed by British Standards Institution (BSI) 1983 incisor classification.

**Exclusion criteria:**

• Craniofacial anomalies, endocrinological disorders, or metabolic bone disease.
• Active periodontal disease or poor oral hygiene based on clinical visual assessment.
• Current smokers; use of NSAIDs or medications affecting salivary gland function.
• Abnormal tooth number or eruption sequence.

Sample size was calculated using G*Power v3.1.9.7 (repeated-measures ANOVA approximation; f = 0.3 [5], α = 0.05, power = 0.85, 3 groups, 3 time points, r = 0.5). Minimum total sample size was 30 patients (10 patients per group); 36 patients were recruited to account for approximately 20% attrition (12 patients per group).

### Orthodontic treatment protocol and elastic prescription

**Fig. 1:**
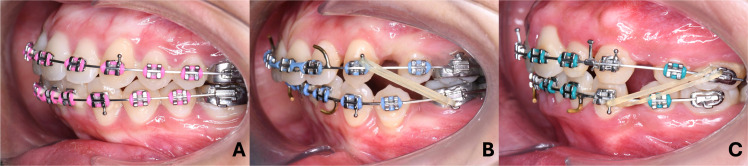
Elastic wear protocol across the three study groups. A - Class I fixed appliance-only reference group: no intermaxillary elastic traction applied throughout the study period. B - Class II elastic-traction group: ¼-inch (6.35 mm), 4.0 oz (113.4 g) elastics (3M™Unitek™) worn bilaterally from maxillary canine hooks to mandibular first molar hooks. C - Class III elastic-traction group: ¼-inch (6.35 mm), 4.0 oz (113.4 g) elastics (3M™ Unitek™) worn bilaterally from maxillary first molar hooks to mandibular canine hooks. Full elastic prescription and compliance protocol are described in the above section.

All participants were treated with pre-adjusted edgewise fixed appliances using the MBT prescription (Master Series® brackets, 0.022 × 0.028-inch slot; American Orthodontics). Recruitment and study initiation commenced after completion of alignment/levelling and placement of 0.019 × 0.025-inch stainless steel working archwires (Ormco) in both arches. Archwires were left passive for one visit prior to baseline saliva collection. The elastic wear protocol for each group is illustrated in Fig. 1.

• Class I group: No intermaxillary elastic traction was applied at any point during the study period. This group received non-extraction fixed appliance treatment only and served as the within-study control for time-dependent biomarker changes attributable to fixed appliance mechanics alone (Fig. 1A).
• Class II group: ¼-inch (6.35 mm), 4.0 oz (113.4 g) latex elastics (3M™Unitek™) worn bilaterally from maxillary canine hooks to mandibular first molar hooks, generating a posteriorly directed force vector on the maxillary arch and an anteriorly directed force vector on the mandibular arch (Fig. 1B). Patients in this group had undergone at least two maxillary premolar extractions prior to study enrolment.
• Class III group: ¼-inch (6.35 mm), 4.0 oz (113.4 g) elastics (3M™ Unitek™) worn bilaterally from maxillary first molar hooks to mandibular canine hooks, generating an anteriorly directed force vector on the maxillary arch and a posteriorly directed force vector on the mandibular arch (Fig. 1C). Patients in this group had undergone at least two mandibular premolar extractions prior to study enrolment.
• Compliance instruction: Full-time wear including during meals; removal only for oral hygiene; verbal and written instructions provided; daily text message reminders sent throughout the study period.

### Study time points

• T_0_ (Baseline): immediately before initiation of intermaxillary elastic traction, following passive archwire placement at the previous visit.
• T_1_ (24 h): after 24 h of elastic wear to capture initial mechanotransduction/lag phase of OTM.
• T_2_ (7 days): after 7 days of continuous elastic wear to capture the osteoclastogenic cascade phase of OTM.

The Class I group provided saliva at corresponding time points to control for time-dependent biomarker changes arising from fixed appliance mechanics alone.

### Saliva collection (Passive drooling method)

Participants were instructed to abstain from eating, drinking, chewing gum, or performing oral hygiene procedures for at least 1 hour before collection [1]. At each time point, they were seated upright and asked to allow unstimulated saliva to pool naturally in the floor of the mouth without swallowing, then to expectorate into a sterile 50 mL polypropylene tube using the passive drooling method until approximately 5 mL had been collected [8]. A timer was used to standardise the sampling duration, which typically required 5 min.

Immediately after collection, all saliva samples were placed on ice, as short-term cooling has been shown to preserve the integrity of salivary proteins without altering proteomic chemistry [9]. Samples were then promptly transported on ice to the laboratory and stored in a freezer at −80 °C, a temperature at which salivary specimens can remain stable for several years with minimal or no detectable biochemical degradation [10]. The samples were not centrifuged prior to freezing, as premature centrifugation has been reported to cause a loss of salivary proteins and compromise downstream proteomic assays [11].

### Saliva processing for downstream analysis

The saliva samples were thawed on ice and subsequently vortexed thoroughly to ensure homogeneity [12]. Then, the saliva samples were centrifuged at 10,000 × g for 5 min at 4 °C using a refrigerated microcentrifuge (Hettich Mikro 220R, Andreas Hettich GmbH & Co. KG, Tuttlingen, Germany) to pellet cellular and particulate material [3]. The pellet was resuspended in 1 mL of cold physiological saline (0.9% sodium chloride) (RinsCap® NS, Malaysia) and vortexed until fully dissolved. A second centrifugation step was performed at 10,000 × g for 5 min at 4 °C, after which the supernatant was removed. This wash procedure (resuspension and centrifugation) was repeated twice more, for a total of three washes, to remove mucins, epithelial debris, microbial contaminants, and residual inhibitors known to interfere with RNA extraction efficiency and qPCR performance. All handling steps were performed promptly at 4 °C to maintain RNA stability and minimise enzymatic degradation. Following the final wash, the resulting purified salivary pellet was immediately processed for RNA extraction.

### RNA extraction

Total RNA was isolated from the washed salivary pellet using the innuPREP RNA Mini Kit 2.0 (IST Innuscreen GmbH, Berlin, Germany), following the manufacturer’s instructions with a modification to the final elution volume. Column‑based RNA purification was chosen because it offers rapid processing, avoids hazardous organic solvents, minimises protein contamination, and is well suited to high‑throughput RT‑qPCR workflows, whereas phenol-chloroform extraction requires handling immiscible organic phases and is more labour‑intensive. Silica-based spin-column systems were used because they provide a rapid and standardised approach for RNA purification while avoiding the hazardous by-products and potential downstream inhibitory effects associated with phenol-chloroform extraction [13].

All preanalytical handling steps were performed promptly at 4 °C to maintain RNA stability and minimise enzymatic degradation. Following the final saline wash, the purified salivary pellet was immediately subjected to RNA extraction using this column-based protocol.

#### Lysis

• Salivary pellet resuspended in 400 µL Lysis Solution RL (innuPREP RNA Mini Kit 2.0) and vortexed briefly.
• Incubated 2 min at room temperature and pipette repeatedly to enhance homogenisation. Incubated a further 3 min to maximise nucleic acid release.
• Lysate transferred onto spin filter and centrifuged at 11,000 × g for 2 min. Collect RNA-containing filtrate.

#### RNA binding

• 400 µL of 70% ethanol was added to the filtrate and mixed gently. (do not vortex).
• Mixture transferred to a different spin filter and centrifuged at 11,000 × g for 2 min, enabling adsorption of nucleic acids onto the silica matrix at the appropriate chaotropic salt conditions.

#### Wash steps

• Wash 1: 500 µL high-salt wash buffer and centrifuged at 11,000 × g for 1 min.
• Wash 2: 700 µL low-salt wash buffer and centrifuged at 11,000 × g for 1 min.
• Additional wash: 80% ethanol and centrifuged at 11,000 × g for 1 min to improve purity.

#### Dry spin

• 3-minute dry spin at 11,000 × g ensured complete evaporation of residual ethanol, as alcohol carry-over can inhibit enzymatic reactions such as reverse transcription.

#### Elution (modified volume)

• 30 µL DNase/RNase-free water was applied to membrane centre and incubated for 1 min at room temperature. Then, centrifuged at 11,000 × g, 1 min to collect the concentrated RNA eluate.
• This reduced elution volume is a column-based optimization strategy to increase RNA concentration.

### RNA quality assessment

RNA concentration and purity were assessed using a NanoDropTM 1000 microvolume spectrophotometer (Thermo Fisher Scientific, Wilmington, DE, USA). One microlitre of each RNA sample was measured at 260, 280 and 230 nm after blanking with DNase/ RNase-free water.

The instrument software automatically calculated the A260-based concentration (using the RNA conversion factor of 40) together with absorbance ratio at 260 and 280 nm (A260/A280) and absorbance ratio at 260 and 230 nm (A260/A230) [14]. Acceptance criteria: A260/A280 = 1.8–2.2; A260/A230 ≥2.0.

Samples below the minimum concentration threshold of 13.3 ng/µL were excluded (insufficient to deliver 200 ng within ≤15 µL maximum input volume in the 20 µL cDNA synthesis reaction).

In the present study, 90 samples from 30 patients met the quality thresholds (mean concentration 277.1 ± 270.1 ng/µL; mean A260/A280 1.96 ± 0.09; mean A260/A230 2.01 ± 0.34). Six patients (13 individual samples) were excluded for insufficient RNA concentration.

### Complementary DNA (cDNA) synthesis

Kit: SensiFAST™ cDNA Synthesis Kit (Cat. No. BIO-65,054; Meridian Bioscience, USA). All reagents were thawed on ice; mix gently; centrifuge briefly before use.

#### Reaction setup (20 µL total volume)

RNA input was standardised to 200 ng to ensure consistency across all samples. Each reverse transcription reaction was prepared to a final volume of 20 µL, comprising:

• 200 ng of total RNA.
• 4 µL 5 × TransAmp™ Buffer (anchored oligo-dT + random hexamers).
• 1 µL Reverse Transcriptase.
• Nuclease-free water to 20 µL.

#### Thermal cycling conditions

• 25 °C for 10 min — primer annealing.
• 42 °C for 15 min — first-strand cDNA synthesis.
• 85 °C for 5 min — reverse transcriptase inactivation.

The cDNA was either used immediately or stored at −20 °C for long-term preservation. The cDNA served as the template for quantitative PCR targeting RANKL, OPG, and the β-actin housekeeping gene, which was used as the internal reference for normalisation of gene expression levels.

### Quantitative real-time PCR (RT-qPCR)

#### Primer sequences

Gene-specific primers for RANKL, OPG, and β-actin were adopted from previously published validated sequences (Table 1). All primers synthesised by Repfon Glamor Sdn. Bhd., Malaysia with the following conditions:

• Working stock: 10 µM; amplicons 80–200 bp; annealing temperature 60 °C.
• Primers were designed to span exon-exon junctions to minimise genomic DNA amplification.

**Table 1:** Primer sequences for RT-qPCR analysis of RANKL, OPG, and β-actin.

Gene Symbol	Encoding Protein	GenBank Accession	Amplicon (bp)	Forward Primer (5′→3′)	Reverse Primer (5′→3′)
RANKL (TNFSF11)	TNF Superfamily Member 11	NM_003701.4	196	5′-ACCAGCATCAAAATCCCAAG-3′	5′-CCCCAAAGTATGTTGCATCC-3′
OPG (TNFRSF11B)	TNF Receptor Superfamily Member 11B	NM_002546.4	185	5′-GGCAACACAGCTCACAAGAA-3′	5′-CGGTAAGCTTTCCATCAAGC-3′
β-Actin (ACTB)	Actin Beta (reference gene)	NM_001101.3	184	5′-AGAGCTACGAGCTGCCTGAC-3′	5′-AGCACTGTGTTGGCGTACAG-3′

#### Reaction setup

Kit: SensiFAST™ SYBR® No-ROX Kit (Cat. No. BIO-98,005; Meridian Bioscience, USA). Instrument: CFX96 Touch Real-Time PCR Detection System (Bio-Rad, USA). Software: CFX Maestro v5.3.022.1030 (Bio-Rad, USA).

All reactions were prepared on ice. Each sample was run in technical duplicate and no-template controls (NTC) were included for each primer pair per plate. The per-reaction composition (20 µL total) were:

• 10 µL 2 × SensiFAST SYBR® No-ROX Master Mix.
• 0.8 µL 10 µM forward primer (final concentration: 400 nM).
• 0.8 µL 10 µM reverse primer (final concentration: 400 nM).
• 1 µL cDNA template (derived from standardised 200 ng RNA input).
• 7.4 µL nuclease-free water

#### Thermal cycling protocol

• Initial denaturation step at 95 °C for 10 min to activate the DNA polymerase.
• 49 amplification cycles of denaturation at 95 °C for 15 s and annealing and extension at 60 °C for 1 min, with fluorescence signal acquisition at the end of each extension step.
• A melt curve analysis was performed from 65 °C to 95 °C with 0.5 °C increments, holding for 5 s at each temperature, with continuous fluorescence acquisition to confirm amplification specificity and exclude primer-dimer formation.
• Melt curve acceptance criterion (specified a priori): single sharp melting peak only. Reactions with multiple peaks or shoulders indicate non-specific amplification and must be excluded.

#### Data analysis: ΔΔCt method and normalisation

Ct values were generated automatically by the CFX Maestro fluorescence threshold algorithm, and the mean Ct of technical duplicates was used for all calculations. Relative expression of RANKL and OPG was quantified using the comparative ΔΔCt method.

• Step 1 — ΔCt: ΔCt = Ct(target gene) − Ct(β-actin). β-actin suitability is confirmed if Ct values are stable across groups and time points (mean 24.27 ± 2.24 cycles in the present study).
• Step 2 — ΔΔCt: ΔΔCt = ΔCt(time point of interest) − ΔCt(T0 baseline). Baseline normalisation is performed within each patient, using their individual T0 value as the reference.
• Step 3 — Fold change = 2^-ΔΔCt, where a fold change of 1.0 indicates expression equivalent to baseline, >1.0 indicates upregulation, and <1.0 indicates downregulation relative to T0.

The RANKL/OPG ratio is calculated from the corresponding fold-change values at each time point. For multi-plate runs, inter-plate normalisation is achieved through the internal β-actin reference gene; ΔΔCt values are calculated relative to T_0_ within each plate. All procedures comply with MIQE guidelines [6].

### Statistical analysis

Software: IBM SPSS Statistics v25.0 (IBM Corp., 2017); GraphPad Prism v10.0.0 for graphical outputs.

**Primary outcomes:**

• RANKL and OPG relative gene expression (fold change, ΔΔCt method).
• RANKL/OPG ratio (calculated from fold-change values at each time point).

Linear mixed models (LMM) were used to analyse the longitudinal repeated-measures dataset. Malocclusion group (Class I/II/III) was modelled as a between-subject fixed effect; time point (T_0_/T_1_/T_2_) as a within-subject fixed effect. The group and time interaction term evaluated whether temporal biomarker trajectories differed between groups. Patient identity was included as a random intercept to account for within-patient correlation across time points. Estimated marginal means (EMM) and 95% confidence intervals were derived from each fitted model. Pairwise post-hoc contrasts were Bonferroni-adjusted (p < 0.05). Effect sizes reported as partial η² (omnibus effects) and Hedges’ g (pairwise contrasts; preferred for small samples). Baseline age and BMI comparability across groups was assessed by Kruskal-Wallis tests.

## Protocol validation

The protocol was validated through application to 90 salivary RNA samples from 30 orthodontic patients (10 per group). RNA extraction yielded a mean concentration of 277.1 ± 270.1 ng/µL (median 178.9 ng/µL; range 13.8–1373.0 ng/µL), with mean A260/A280 of 1.96 ± 0.09 and mean A260/A230 of 2.01 ± 0.34, consistent with high-quality nucleic acids suitable for RT-qPCR. All 90 included samples exceeded the minimum concentration threshold of 13.3 ng/µL. 13 samples from 6 patients were excluded at the RNA quality assessment stage as the RNA concentration was insufficient for reliable reverse transcription, and these patients were omitted from all subsequent cDNA synthesis, RT‑qPCR, and statistical analyses.

β-actin demonstrated stable Ct values across study groups and all three time points (mean 24.27 cycles; SD 2.24 cycles), confirming its suitability as an internal reference for normalisation of RANKL and OPG gene expression in this cohort. Melt curve analysis confirmed single, sharp melting peaks for all included RANKL and OPG reactions. No-template controls showed no amplification, excluding reagent contamination and primer-dimer artefacts.

## Limitations

• RNA yield from unstimulated whole saliva is inherently variable (wide inter-individual range). In the present study, 16.7% of enrolled participants were excluded at the RNA quality control stage. Investigators should recruit additional participants (recommended ≥20% buffer above the target per-protocol sample size) to account for this attrition.
• Operator blinding was not possible in the single-investigator design. Bias was minimised through sample coding, automated Ct acquisition, and a priori-defined melt curve inclusion criteria. Future multi-investigator replication studies should implement formal separation of clinical and laboratory roles.
• Salivary flow rate was not formally quantified per participant at each session; absolute RNA and biomarker concentrations may carry residual inter-individual variability attributable to flow rate differences.
• A single internal reference gene (β-actin) was used for normalisation. Use of at least two reference genes is recommended by MIQE guidelines where feasible; validation of additional stable housekeeping genes for salivary orthodontic biomarker studies is encouraged in future work.
• Periodic compliance with elastic wear between appointments was not independently verified by objective monitoring tools; self-reported and clinically observed compliance was relied upon.

## Declaration of competing interest

The authors declare that they have no known competing financial interests or personal relationships that could have appeared to influence the work reported in this paper.

## Data Availability

Data will be made available on request.
